# Interbacterial Transfer of Carbapenem Resistance and Large Antibiotic Resistance Islands by Natural Transformation in Pathogenic Acinetobacter

**DOI:** 10.1128/mbio.02631-21

**Published:** 2022-01-25

**Authors:** Anne-Sophie Godeux, Elin Svedholm, Samuel Barreto, Anaïs Potron, Samuel Venner, Xavier Charpentier, Maria-Halima Laaberki

**Affiliations:** a Centre International de Recherche en Infectiologie (CIRI), INSERM, U1111, Université Claude Bernard Lyon 1, CNRS, UMR5308, École Normale Supérieure de Lyon, Université de Lyon, Villeurbanne, France; b Université de Lyon, VetAgro Sup, Marcy l’Etoile, France; c UMR CNRS 5558, Laboratoire de Biométrie et Biologie Évolutive (LBBE), Université Claude Bernard Lyon 1, Villeurbanne, France; d French National Reference Center for Antibiotic Resistance, University Hospital of Besançon, Besançon, France; e UMR6249, CNRS Chrono-Environnement, Franche-Comté University, Besançon, France; Louis Stokes Veterans Affairs Medical Center

**Keywords:** AbaR, *Acinetobacter baumannii*, OXA23, type VI secretion system, antibiotic resistance, carbapenem resistance, horizontal gene transfer, natural transformation, resistance island

## Abstract

Acinetobacter baumannii infection poses a major health threat, with recurrent treatment failure due to antibiotic resistance, notably to carbapenems. While genomic analyses of clinical strains indicate that homologous recombination plays a major role in the acquisition of antibiotic resistance genes, the underlying mechanisms of horizontal gene transfer often remain speculative. Our understanding of the acquisition of antibiotic resistance is hampered by the lack of experimental systems able to reproduce genomic observations. We here report the detection of recombination events occurring spontaneously in mixed bacterial populations and which can result in the acquisition of resistance to carbapenems. We show that natural transformation is the main driver of intrastrain but also interstrain recombination events between A. baumannii clinical isolates and pathogenic species of Acinetobacter. We observed that interbacterial natural transformation in mixed populations is more efficient at promoting the acquisition of large resistance islands (AbaR4 and AbaR1) than when the same bacteria are supplied with large amounts of purified genomic DNA. Importantly, analysis of the genomes of the recombinant progeny revealed large recombination tracts (from 13 to 123 kb) similar to those observed in the genomes of clinical isolates. Moreover, we highlight that transforming DNA availability is a key determinant of the rate of recombinants and results from both spontaneous release and interbacterial predatory behavior. In the light of our results, natural transformation should be considered a leading mechanism of genome recombination and horizontal gene transfer of antibiotic resistance genes in Acinetobacter baumannii.

## INTRODUCTION

Acinetobacter baumannii is a Gram-negative bacterium responsible for a wide range of infections in both humans and animals (1, 2). This multidrug-resistant (MDR) agent poses a health threat, particularly in intensive care units, where it can lead to bacteremia and ventilator-associated pneumonia. Consequently, secondary infections with multidrug-resistant A. baumannii have been reported during the COVID-19 pandemic (3, 4). A. baumannii infections are frequently resistant to multiple antibiotics, including to carbapenems. For Europe only, a combined resistance to fluoroquinolones, aminoglycosides, and carbapenems is observed for nearly 30% of invasive Acinetobacter sp. isolates (5). In A. baumannii, carbapenem resistance is mainly associated with the expression of OXA23 carbapenemase, encoded by the *bla*_OXA-23_ gene (6). The composite transposon Tn*2006*, formed by two ISAba1 insertion sequences framing the *bla*_OXA-23_ gene, is the main genetic context for the *bla*_OXA-23_ gene and can be found in different locations, including plasmids, but it is most often found inserted in the large resistance island AbaR4 (6, 7). Such large and diverse A. baumannii resistance islands (Ab-RI) are potential contributors to the multidrug resistance phenotype observed in A. baumannii. The first description of an Ab-RI was reported in 2006 in the epidemic strain AYE with the AbaR1 island consisting in an 86-kb-long genomic structure (8). Since then, analysis of more than 3,000 A. baumannii genomes revealed that Ab-RI are present in nearly 65% of them (9). These genomic islands present a great diversity in gene content, with often multiple putative antibiotic resistance genes (10, 11). Ab-RI were presumed to be initially acquired through plasmid conjugation followed by chromosomal insertion and to evolve through multiple insertions and rearrangements of insertion sequences (12, 13). However, genome analyses also support their horizontal transfer between distantly related isolates, as exemplified by the acquisition of the 21-kb-long AbGRI3 resistance island (14) and the 35-kb-long AbGRI5 (15). Both acquisitions involved large recombination events (up to 34 kb-long) at the sequence flanking the island. Indeed, high rates of genome recombination are a hallmark of A. baumannii genomes (16, 17). Recombination events may have led to the acquisition by MDR strains of *parC* or *gyrA* alleles conferring resistance to fluoroquinolones and of IS*Aba1* upstream of the *ampC* gene, leading to resistance to 3rd-generation cephalosporins (14, 17, 18). Genome analysis of isolates from a longitudinal study also offered a glimpse of the gene transfer and recombination going on in the hospital setting, with the acquisition of the *bla*_OXA-72_ variant of the *bla*_OXA24/40_-type carbapenemase gene and recombination affecting the *bla*_OXA-51_ locus (19).

Despite their importance in the evolution of A. baumannii into an MDR pathogen, the mechanisms leading to genome recombination and recombination-dependent acquisition of resistance genes and Ab-RI remain elusive. Indeed, few studies have experimentally investigated the horizontal transfer of chromosomal antibiotic resistance genes in A. baumannii. The chromosomal transfer of an 11-kb-long Tn*215* harboring *bla*_NDM-1_ during mating of isolate R2090 to the reference strain CIP 70.10 was experimentally observed and involved the acquisition of a 65-kb region through homologous recombination (20). While transduction by prophages was suggested, the mechanism of transfer was not elucidated. Phage particles, present in prepared fractions from culture supernatant of the clinical isolate NU-60, were found to mediate chromosomal transfer to the reference strain ATCC 17978 (21). If generalized transduction by strain-specific prophages is a potential mechanism of resistance gene transmission, natural transformation is another potential and highly conserved route. Natural transformation allows bacteria to actively import exogenous DNA, and provided there is sufficient sequence identity, it integrates the recipient cell’s genome by homologous recombination (22). Most A. baumannii isolates and also closely related Acinetobacter nosocomialis were found to be capable of natural transformation when presented with purified DNA (23–27). Yet we currently have a limited understanding of the role of natural transformation in genome dynamics and the spread of antibiotic resistance in A. baumannii populations.

In the current study, we explored the intraspecies and intragenus transfer of antibiotic resistance, notably to carbapenems, which is spontaneously occurring in mixed populations of pathogenic Acinetobacter spp. We provide evidence that natural transformation is the main transfer route and fosters recombination events and the acquisition of multiple resistance genes carried by large genomic island. Our results obtained experimentally replicate the large recombination events observed in A. baumannii chromosomes and suggest a major role played by natural transformation in the dynamics of A. baumannii genomes.

## RESULTS

### Intra- and interspecies recombinants are produced in mixed populations of pathogenic Acinetobacter.

**FIG 1:**
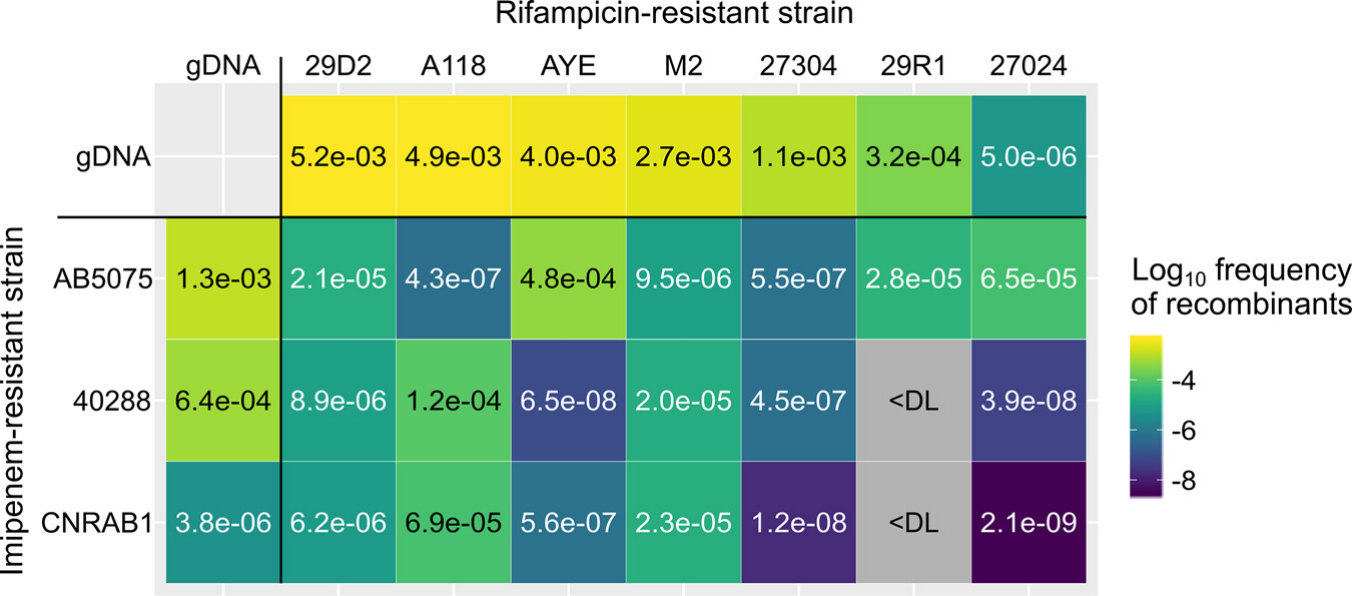
Recombinants are produced in mixed cultures of A. baumannii and A. nosocomialis. Bacterial suspensions of rifampicin-resistant (Rif^r^) isolates of carbapenem-sensitive strains (29D2, A118, AYE, M2, 27304, 29R1, and 27024) were mixed with the carbapenem-resistant (Imi^r^) clinical isolates AB5075, 40288, and CNRAB1 using cultures adjusted to an OD_600_ of 0.01. The mixture (2.5 μL) was deposited on the surface of tryptone-NaCl medium and incubated overnight at 37°C. Rif^r^ and Imi^r^ recombinants were determined after 24 h of mixed culture. Recombinant frequencies represent the ratio of Rif^r^ and Imi^r^ CFU over the total CFU counts. Frequency of recombinants is presented as a heat map with indicated average frequencies from two or three biological replicates with three technical replicates each. <DL, below detection limit (∼10^−9^). Top row and outermost left column show the transformation frequency displayed under the same condition by the unmixed isolates, tested by incubating the original isolates with genomic DNA (gDNA) extracted from their Rif^r^ derivatives and select with rifampicin. The transformation frequencies (ratio of Rif^r^ CFU by total CFU counts) are represented by a color gradient from blue to yellow. Data shown are the means of natural transformation assays done in two or three biological replicates with three technical replicates each.

To test the possible transfer of antibiotic resistance in bacterial populations, we first assessed if recombinants could arise in mixed cultures of isolates harboring distinct resistance determinants. Three imipenem-resistant (Imi^r^) A. baumannii clinical isolates (AB5075, 40288, and CNRAB1) and seven imipenem-sensitive (Imi^s^) isolates of either A. baumannii (29D2, A118, AYE, 27304, 29R1, and 27024) or A. nosocomialis (M2) were selected. Rifampicin-resistant (Rif^r^) mutants of the Imi^s^ isolates were obtained. Each of these imipenem-sensitive and Rif^r^ isolates was grown for 24 h in mixed culture with each Imi^r^ isolate. The frequency of Rif^r^ Imi^r^ recombinants in the mixed cultures was then determined. Rif^r^ Imi^r^ recombinants were detected in 19 of the 21 tested combinations, with highly variable frequencies ranging from 2.10 × 10^−9^ to 4.82 × 10^−4^ (Fig. 1). Importantly, an imipenem-sensitive isolate did not become Imi^r^ in the absence of an Imi^r^ isolate. And similarly, in the absence of Rif^r^ isolates, Imi^r^ isolates spontaneously develop resistance to rifampicin at frequencies below the detection limit of this assay (10^−9^). This strongly suggests that Rif^r^ Imi^r^ recombinants result from horizontal gene transfer (HGT) between the mixed isolates. All tested isolates were capable of natural transformation under the tested growth conditions when presented with purified genomic DNA (gDNA) extracted from their own Rif^r^ derivative (Fig. 1). We could not correlate transformation efficiency with nucleotide relatedness, as most pairs had comparable nucleotide identities (around 98% [see Fig. S1 in the supplemental material]), with the exception of the pairing with A. nosocomialis strain M2, which had the lowest nucleotide identities (around 92%) but high recombinant frequencies. Most isolates showed high transformability (transformation frequencies > 1 × 10^−3^), while two isolates (CNRAB1 and 27024) presented lower transformation frequencies (1 × 10^−6^). Pairing these two isolates generated only a few recombinants, while pairing with a more transformable isolate generated up to 10,000 times more recombinants, suggesting that at least one of the two isolates must be transformable to obtain recombinants. Yet some combinations of highly transformable isolates were poorly productive in recombinants (40288 × AYE), suggesting that factors other than the intrinsic transformability of isolates play a role in the production of recombinant progeny.

10.1128/mBio.02631-21.1 FIG S1 Recombinant frequency as a function of genomic relatedness between paired strains. Analysis of recombinant frequencies for paired cotransformants as presented in Fig. 1 in the main text depending on the pairwise average nucleotide identity (ANI). For each pairing, the imipenem-resistant strain is highlighted in panel A and the rifampicin-resistant one in panel B. Download FIG S1, TIF file, 0.4 MB. Copyright © 2022 Godeux et al. 2022 Godeux et al. https://creativecommons.org/licenses/by/4.0/ This content is distributed under the terms of the Creative Commons Attribution 4.0 International license.

### Pathogenic Acinetobacter rapidly acquire carbapenem resistance by natural transformation in mixed culture.

**FIG 2:**
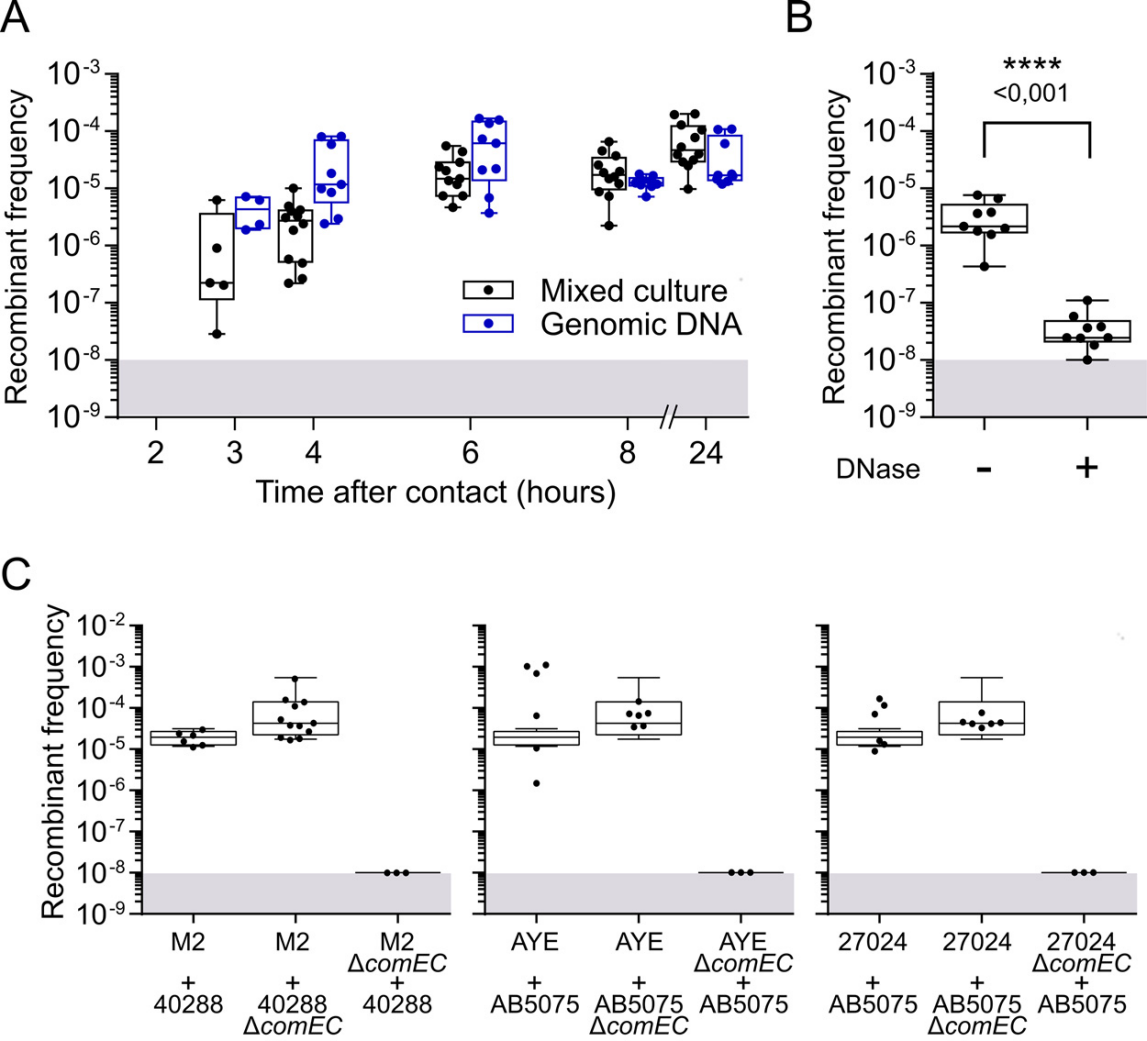
Acquisition of carbapenem resistance by natural transformation in a mixed culture. (A) Kinetics of emergence of carbapenem- and rifampicin-resistant recombinants in a mixed culture of M2 Rif^r^ and 40288 (Imi^r^). Bacterial suspensions of M2 Rif^r^ at an OD_600_ of 0.01 were mixed with either an equal volume of a suspension of 40288 at an OD_600_ of 0.01 or gDNA extracted from 40288 (at 200 ng/μL). The mixture (2.5 μL) was deposited on the surface of tryptone-NaCl medium and incubated overnight at 37°C. Recombinant frequencies represent the ratio of Rif^r^ and Imi^r^ CFU over the total. The limit of detection (10^−8^) is indicated by the gray area. (B) Sensitivity of recombinant frequencies to DNase I treatment under the same conditions as for panel A, with a mixed culture of 6 h. (C) Recombinants emerge by the ComEC-dependent acquisition of carbapenem resistance by susceptible isolates. Rif^r^ isolates of M2, AYE, 27024, or their *comEC* mutants were mixed with the carbapenem-resistant (Imi^r^) clinical isolates 40288 and AB5075 or their *comEC* mutants. Rif^r^ and Imi^r^ recombinants were determined after 24 h of mixed culture. The boxplots represent the distributions of Rif^r^ and Imi^r^ recombinants divided by the total CFU count (recombinant frequencies). A horizontal line represents the median. The limit of detection (10^−8^) is indicated by the gray area. In panel B, statistical analysis was conducted using the nonparametric Mann-Whitney-Wilcoxon test (two tailed). *P* values are indicated.

The conditions under which we observed the rise of recombinants are permissive to natural transformation, yet we could not exclude other mechanisms of horizontal gene transfer. Natural transformation was previously reported to occur during exponential growth of A. baumannii (27, 28). Therefore, we sought to determine whether the kinetics of increasing recombinant numbers under mixed culture are consistent with a role of natural transformation. Focusing on the M2 × 40288 combination of two pathogenic Acinetobacter strains, we could detect recombinants as early as 3 h after mixing the two isolates (Fig. 2A). The frequency of recombinants reached a plateau at 6 h. The kinetics of recombinant emergence were strikingly similar when we provided the M2 isolate with genomic DNA of 40288, further indicating that recombinants emerge by natural transformation (Fig. 2A). Moreover, in a 4-h-long mixed-culture experiment, addition of DNase I reduced the size of the recombinant population by 2 orders of magnitude, ruling out the role of phages or outer membrane vesicles in recombinant production (Fig. 2B). To further validate the role of natural transformation, we tested the emergence of recombinants with Δ*comEC*::*aacC4* derivatives, deficient in DNA import. Combination of the M2 strain with the 40288 Δ*comEC* mutant did not alter the level of recombinants (Fig. 2C). However, inactivation of *comEC* in the M2 strain completely abolished recombinants production indicating that the recombinants are formed from M2 bacteria that have acquired carbapenem resistance by natural transformation. Consistent with the role of natural transformation, acquisition of carbapenem resistance in mixed culture requires functional type IV pili and recombination machinery, as M2 Δ*pilA*::*kan* and Δ*recA*::*aacC4* derivatives did not produce any recombinants (Fig. S2). Similarly, the results demonstrate that the A. baumannii strains AYE and 27024 use natural transformation to acquire carbapenem resistance from AB5075 (Fig. 2C). Interestingly, carbapenem-resistant transformants of 27024 are produced at higher rates than the transformation rate of 27024 when tested with its own gDNA, conferring resistance to rifampicin (Fig. 1). This suggests that acquisition of carbapenem resistance is more effective than the acquisition of the mutation in the *rpoB* gene, conferring resistance to rifampicin. The mutations in this housekeeping gene are probably highly costly in terms of fitness (29, 30), which may explain the absence of recombinants resulting from the acquisition of the rifampicin resistance by transformable carbapenem-resistant isolates. In conclusion, in mixed populations of Acinetobacter, carbapenem resistance can rapidly and efficiently spread to susceptible isolates by natural transformation.

10.1128/mBio.02631-21.2 FIG S2 Recombinants emerge in mixed culture in a PilA- and RecA-dependent manner. Rifampicin-resistant (Rif^r^) isolates of M2 or its *pilA*::*kan* or *recA*::*aacC4* mutant were mixed with the carbapenem-resistant (Imi^r^) clinical isolate 40288 Δc*omEC* mutant. Rif^r^ and Imi^r^ recombinants were determined after 24 h of mixed culture. The boxplots represent the distributions of Rif^r^ and Imi^r^ recombinants divided by the total CFU count (recombinant frequencies). A horizontal line represents the median. The limit of detection (10^−8^) is indicated by the gray area. Download FIG S2, TIF file, 0.1 MB. Copyright © 2022 Godeux et al. 2022 Godeux et al. https://creativecommons.org/licenses/by/4.0/ This content is distributed under the terms of the Creative Commons Attribution 4.0 International license.

### Chromosomal DNA transfer is stimulated by T6SS-killing activity but primarily relies on contact-independent spontaneous release of DNA.

**FIG 3:**
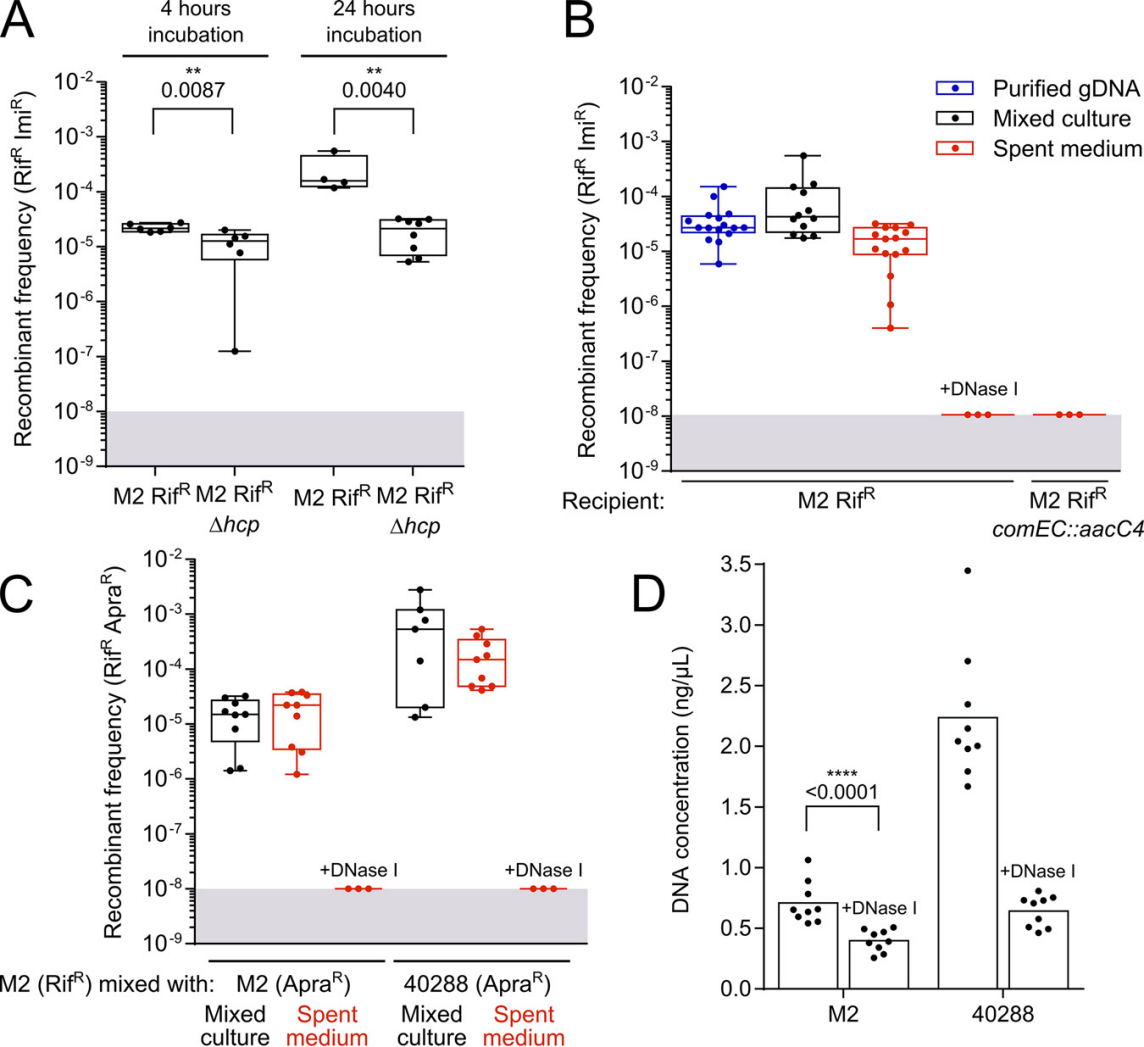
Contribution of contact-dependent T6SS-killing activity and contact-independent DNA release to the acquisition of the carbapenem resistance in mixed cultures. (A) Contribution of the T6SS to the frequency of recombinants generated in a mixed culture of 40288 and M2 Rif^r^. 40288 was mixed with the M2 Rif^r^ strain or the M2 Rif^r^ Δ*hcp* mutant, defective for T6SS activity. Imipenem-resistant transformants of the M2 strain were determined after 4 h or 24 h of mixed culture. Transformation frequencies represent the ratio of M2 Rif^r^ Imi^r^ CFU over the total count of M2 Rif^r^ CFU. (B) Contribution of contact-independent DNA release by 40288 to the acquisition of the imipenem resistance by M2 in mixed culture. Bacterial suspensions of M2 Rif^r^ at an OD_600_ of 0.01 were mixed with an equal volume of either genomic DNA extracted from 40288 (at 200 ng/μL), a suspension of 40288 at an OD_600_ of 0.01, or filtered spent medium of a 4-h culture in tryptone-NaCl medium of 40288. The mixture (2.5 μL) was deposited on the surface of tryptone-NaCl medium and incubated for 6 h at 37°C. Recombinant frequencies represent the ratio of Rif^r^ and Imi^r^ CFU over the total. (C) Contact-independent DNA release promotes both intra- and interstrain recombination. The frequency of intrastrain recombinants from M2 Rif^r^ and M2 Apra^r^ strains was determined in mixed culture or when M2 Rif^r^ was exposed to the spent medium of the M2 Apra^r^ strain. To test for interstrain recombination, M2 Rif^r^ was mixed with 40288 Apra^r^ or with the spent medium from that strain. Recombinant frequencies represent the ratio of Rif^r^ and Apra^r^ CFU over the total. (D) Fluorescence-based quantification of DNA present in the spent medium of strains M2 and 40288. Samples treated with DNase I represent the detection limit of the assay (∼0.4 ng). When displayed, *P* values of statistical analysis were obtained using the nonparametric Mann-Whitney-Wilcoxon test (two tailed). The limit of detection (10^−8^) is indicated by the gray area.

The essential role of natural transformation in the acquisition of carbapenem resistance suggests that chromosomal DNA of the carbapenem-resistant isolate is released during mixed culture. In Vibrio cholerae, the type VI secretion system (T6SS) mediates the killing of nonkin cells, promoting the release of DNA from prey cells, which is then imported by natural transformation by the predatory bacteria (31). Acinetobacter baylyi also produces a T6SS to kill prey, allowing the uptake of their DNA (32). In order to determine the contribution of T6SS-dependent killing to the chromosomal DNA transfer in the Acinetobacter mixed culture, we examined the combination of strains 40288 and M2, the latter being known for producing an active T6SS in LB medium (33). Under these conditions, we confirmed that M2 efficiently kills the 40288 strain, with a reduction of the population of 40288 by 4 orders of magnitude (Fig. S3A). A Δ*hcp* mutant of the M2 strain could not kill 40288, demonstrating that killing is indeed T6SS dependent (Fig. S3A). However, under the condition in which we observed transformation-dependent chromosomal transfer, M2 T6SS is presumably poorly active, with comparable viable 40288 cells titers when mixed for 4 h with either M2 or its Δ*hcp* derivative (Fig. S3B). Yet inactivation of M2 T6SS slightly lowered the rate of recombinants at 4 h, and reduced it by 1 order of magnitude at 24 h (Fig. 3A). This indicates that residual T6SS activity contributes to DNA release and chromosomal DNA acquisition by the M2 strain. Yet even in the absence of a functional T6SS, recombinant frequency remained high (10^−5^). We thus hypothesized that DNA is also released independently of contact-dependent killing mediated by the T6SS. To test this hypothesis, we collected culture supernatant of 40288 at 4 h in the absence of M2. The M2 strain presented with this spent medium could acquire carbapenem resistance almost as efficiently as when grown with 40288 or when presented with purified genomic DNA from 40288 (Fig. 3B). No carbapenem-resistant recombinant was observed when the spent medium was treated with DNase I or if the M2 strain was inactivated for DNA uptake (Δ*comEC*). This demonstrated that contact-independent DNA release is sufficient to promote chromosomal DNA acquisition by nonkin cells.

10.1128/mBio.02631-21.3 FIG S3 T6SS-dependent killing activity of the M2 strain on the 40288 isolate in LB medium and under conditions of mixed culture. Recovery of the A. baumannii strain 40288 incubated either with the wild-type M2 strain (WT) or with its *hcp* gene mutant derivative (Δ*hcp*) is shown. (A) Cocultivation of 40288 with the M2 strain during in LB medium as described previously (1) resulted in a 4-log decrease of the 40288 population, while inactivating the T6SS of M2 (Δ*hcp*) abolished the killing activity. (B) In the mixed-culture setup used to observe the rise of recombinants, mixed culture of 40288 with M2 for 4 h did not significantly reduce the population size of 40288. Download FIG S3, TIF file, 0.4 MB. Copyright © 2022 Godeux et al. 2022 Godeux et al. https://creativecommons.org/licenses/by/4.0/ This content is distributed under the terms of the Creative Commons Attribution 4.0 International license.

Given that DNA release is independent of the presence of another strain, DNA release and transformation may also occur between individuals of the same strain. We tested this possibility by examining the ability of M2 Rif^r^ individuals to acquire the apramycin resistance (Apra^r^) marker from an M2 Δ*comEC* mutant. In this mixed-culture setup, Rif^r^ Apra^r^ recombinants occurred at a frequency of 10^−5^ (Fig. 3C). The same frequency was observed when using spent medium of the M2 Apra^r^ Δ*comEC* mutant to transform M2 Rif^r^. Together, these findings show that M2 cells spontaneously release DNA that can serve to transform other cells of the population. Yet in a mixed-culture setup, M2 acquired the apramycin resistance marker from 40288 Δ*comEC* at higher rates than from itself (M2 Δ*comEC*) (Fig. 3C). The same result was obtained with the spent medium, raising the possibility that the DNA released by 40288 was more potent in producing transformants. As M2 can be transformed with the same efficiency with either M2 or 40288 gDNA (Fig. S4), we hypothesized that 40288 simply releases more DNA than the M2 strain. Indeed, quantification of DNA in spent medium from 4-h cultures showed that 40288 released at least 3 times more DNA than M2 (Fig. 3D). Yet surprisingly, quantities of DNA in the spent media remained very low, with concentrations of around 2 ng/μL for 40288 strain and 0.7 ng/μL for M2 strain, almost as low as the detection limit (0.4 ng/μL, obtained with DNase I-treated samples). However, this small amount collected at 4 h of growth was sufficient to generate transformants at frequencies comparable to those obtained with about 100-times-larger amounts of purified gDNA in a 6-h window (Fig. 3B). Overall, our results indicate that the amount of spontaneously released DNA is the major determinant of the rate of recombinants generated from individuals of the same or different strains. In conclusion, the T6SS-dependent and contact-independent release of small amounts of genomic DNA by pathogenic Acinetobacter fuels horizontal gene transfer and the acquisition of antibiotic resistance genes.

10.1128/mBio.02631-21.4 FIG S4 Strain M2 of A. nosocomialis is equally transformed with DNA from itself and from the distantly related strain 40288 of A. baumannii. Strain M2 was incubated under transformation conditions (>16 h) with genomic DNA extracted either from an M2 Δ*comEC*::*aacC4* (Apra^r^) or from a 40288 Δc*omEC*::*aacC4* (Apra^r^) strain. Transformation frequency represents the CFU count on an apramycin plate divided by the total CFU count on nonselective plates. Download FIG S4, TIF file, 0.1 MB. Copyright © 2022 Godeux et al. 2022 Godeux et al. https://creativecommons.org/licenses/by/4.0/ This content is distributed under the terms of the Creative Commons Attribution 4.0 International license.

### Acquisition of resistance can result from the horizontal transfer of large genomic islands.

To characterize the chromosomal transfer of the imipenem resistance at a genetic level, we sought to identify the imipenem resistance determinant in the 40288 donor strain and in transformants. 40288 is a carbapenem-resistant strain isolated from a diseased animal and carries the *bla*_OXA-23_ carbapenem resistance gene (34). An initial assembly of the 40288 genome using Illumina reads resulted in 54 contigs, and the *bla*_OXA-23_ carbapenem resistance gene was found in a 2.4-kb contig whose exact location in the genome remained unresolved. A hybrid assembly combining the Illumina reads with long reads (Oxford Nanopore technology) resulted in the complete genome of 40288 showing a single circular contig of 4,084 kb. The genome revealed the presence of the *bla*_OXA-23_ gene as part of the 4.8-kb Tn*2006* transposon inserted at two distinct locations. One Tn*2006* transposon is within the large AbaR4 island of 16 kb inserted into the *comM* gene. We previously demonstrated that in A. baumannii the presence of a resistance island in this gene reduces the transformation level (35). Consistently, acquisition of AbaR4 into the *comM* gene subsequently lowers the transformation level of the recombinant strains 10-fold (Fig. S5A). The other copy of Tn*2006* is inserted into the beginning of the *vgrG* gene, encoding a T6SS component. This gene, in a putative operon with a LysM domain effector, is hereafter referred as *vgrG3* (36). Insertion of Tn*2006* into the *vgrG3* gene did not impair T6SS-mediated killing, as introduction of this insertion in the *vgrG3* gene of the M2 strain did not alter its T6SS-medated killing of Escherichia coli and A. baumannii, in contrast to the case with a Δ*hcp* mutant (Fig. S5B and C).

10.1128/mBio.02631-21.5 FIG S5 Phenotypic outputs of acquisition of AbaR4 and Tn*2006* from strain 40288. (A) Acquisition of AbaR4 in the *comM* gene lowers the transformation levels of the recipient strain. Bacterial suspensions of M2 Rif^r^ or two of its derivatives that acquired AbaR4 in the *comM* gene in previous mixed-culture experiments were mixed with an equal volume of 40288 Δ*comEC*::*aacC4*, all strains at an OD_600_ of 0.01. The mixture (2.5 μL) was deposited on the surface of transformation medium. Recombinant frequency represents the ratio of M2 Rif^r^ Apra^r^ CFU over the total count of Rif^r^ CFU. *P* values of statistical analysis were obtained using the nonparametric Mann-Whitney-Wilcoxon test (two tailed). The limit of detection (10^−8^) is indicated by the gray area. (B) T6SS-mediated killing assay of a VgrG3 impaired mutant of A. nosocomialis strain M2 in LB medium. Killing assays were performed as described for Fig. S1A. Escherichia coli strain JW2912 (Kan^r^) and A. baumannii strain 40288 were both used as prey and were incubated either with the wild-type M2 strain, its *hcp* gene mutant derivative (Δ*hcp*), or an imipenem-resistant transformant carrying an insertion into the *vgrG3* gene (*vgrG3*::Tn*2006*; two transformants tested). Download FIG S5, TIF file, 0.6 MB. Copyright © 2022 Godeux et al. 2022 Godeux et al. https://creativecommons.org/licenses/by/4.0/ This content is distributed under the terms of the Creative Commons Attribution 4.0 International license.

**FIG 4:**
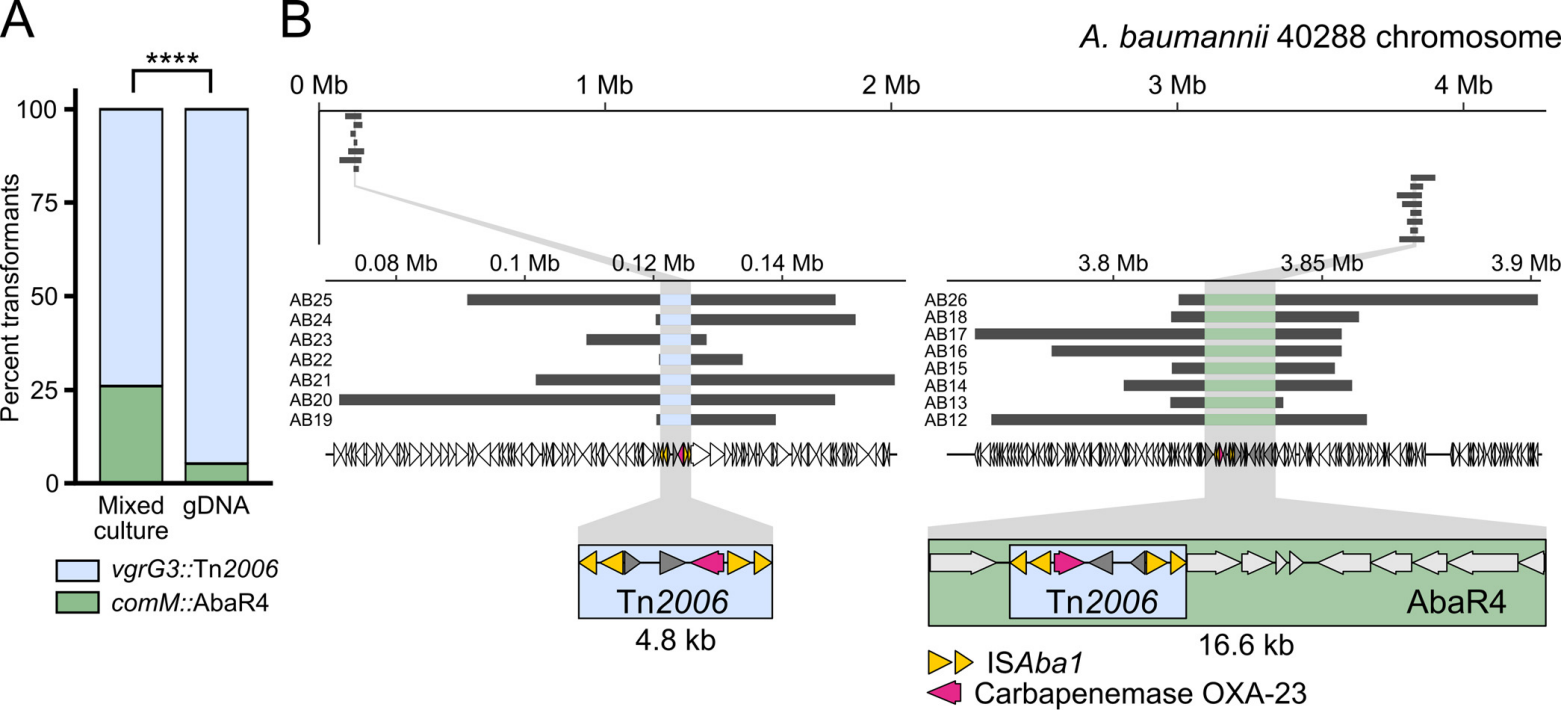
Genomic analysis of the acquisition of imipenem resistance. (A) PCR-based analysis of the chromosomal location of the Tn*2006* transposon and associated *bla*_OXA-23_ gene, conferring resistance to imipenem. One hundred Imi^r^ and *bla*_OXA-23_ PCR-positive CFU from 2 independent transformation assays were analyzed by PCR probing insertion in the *comM* gene. Statistical significance was calculated using Pearson’s chi-squared test returning a *P* value of <0.0001 (****). (B) Graphical representation of the chromosome of the 40288 strain (thin black line) and location of the acquired DNA fragments (light gray thick lines) by transformants of the A. nosocomialis strain M2 during mixed culture with 40288. Bottom left and right diagrams represent close-ups of the regions in which the *bla*_OXA-23_ gene is acquired as part of the Tn*2006* element (blue box) in the *vgrG3* gene and as part of the Tn*2006* element within the AbaR island (green box) inserted in the *comM* gene. Acquired regions were determined by sequencing the genomes of imipenem-resistant recombinants of M2 which had acquired *bla*_OXA-23_ on a NovaSeq instrument. Variant calling and identification of converted markers (SNPs of 40288 acquired by M2) were used to delineate the acquired regions (see Materials and Methods).

Importantly, we found that either copy of *bla*_OXA-23_ confers the same level of resistance to imipenem on the M2 strain (Table S2). However, the rate of their horizontal transfer may be affected by their genetic context. Acquisition of the *bla*_OXA-23_ gene from *vgrG3*::Tn*2006* requires incorporation of 4.8 kb of heterologous sequence, while acquisition of the other copy would involve acquisition of the whole AbaR4 island of 16.6 kb. When genomic DNA from 40288 is provided to the M2 strain, the vast majority of imipenem-resistant transformants results from the acquisition of *vgrG3*::Tn*2006*, with only 5% of transformants acquiring the 16-kb-long AbaR4 island in *comM* (Fig. 4A). In contrast, under the mixed-culture condition, this percentage increased 5-fold (Fig. 4A), suggesting that the mixed culture allowed the acquisition of large DNA fragments. To further determine the length of imported DNA molecules under these conditions, we characterized by whole-genome sequencing the extent of DNA recombination which had occurred in the M2 transformants. To this end, 15 imipenem-resistant transformants of M2 obtained from mixed cultures with 40288 were analyzed: 7 resulting from the acquisition of the *vgrG3*::Tn*2006* insertion and 8 from the acquisition of *comM*::AbaR4. We used the nearly 300,000 single nucleotide polymorphisms (SNPs) between the two strains to identify the parts of the 40288 genome which had been incorporated into the M2 genome (Fig. S6). The succession of acquired SNPs revealed discontinuous recombination tracts on each side of the acquired heterologous sequence (Fig. S6). The discontinuous tracts most likely reflect multiple strand invasion events from the same imported DNA molecule (37). We thus used the position of the outermost acquired SNP to estimate the length of the DNA molecule of 40288 which was imported by the M2 recipients (Fig. 4B). We estimated that recombinants which have acquired Tn*2006* in *vgrG3* had imported DNA fragments ranging from 13 to 77 kb, while those acquiring AbaR4 had imported DNA fragments ranging from 27 to 89 kb (Table S3). Thus, transformants resulted from the recombination of DNA molecules much larger than the acquired heterologous sequences. This suggests that the mixed culture is prone to allow the acquisition of large heterologous sequences such as those of genomic islands.

10.1128/mBio.02631-21.6 FIG S6 Genomic analysis of the acquisition of imipenem resistance. Graphical representation of the chromosome of the 40288 strain (boxed area) and location of the acquired DNA fragments (light gray thick lines) by transformants of A. nosocomialis strain M2 during mixed culture with 40288. Top and bottom panels represent close-ups of the regions in which the *bla*_OXA-23_ gene is acquired as part of the Tn*2006* element (top box) in the *vgrG3* gene and as part of the Tn*2006* element within the AbaR island (bottom box) inserted in the *comM* gene. Acquired regions were determined by sequencing the genomes of imipenem-resistant recombinants of M2 which had acquired *bla*_OXA-23_ on a NovaSeq instrument. Variant calling and identification of converted markers (SNPs of 40288 acquired by M2) were used to delineate the acquired regions (see Materials and Methods). Thin green lines represent coverage, and the outermost right scale indicates the results of read screening corresponding to the AbaR sequence in recombinants. Orange triangles represent individual SNPs used to determine recombination tracts. Download FIG S6, TIF file, 0.5 MB. Copyright © 2022 Godeux et al. 2022 Godeux et al. https://creativecommons.org/licenses/by/4.0/ This content is distributed under the terms of the Creative Commons Attribution 4.0 International license.

10.1128/mBio.02631-21.9 TABLE S2 Antimicrobial susceptibility profiles of mixed-culture transformants. Download Table S2, PDF file, 0.03 MB. Copyright © 2022 Godeux et al. 2022 Godeux et al. https://creativecommons.org/licenses/by/4.0/ This content is distributed under the terms of the Creative Commons Attribution 4.0 International license.

10.1128/mBio.02631-21.10 TABLE S3 Estimated length of the imported DNA molecules leading to the detected recombination tracts. **REFERENCE** 1. Fournier P-E, Vallenet D, Barbe V, Audic S, Ogata H, Poirel L, Richet H, Robert C, Mangenot S, Abergel C, Nordmann P, Weissenbach J, Raoult D, Claverie J-M. 2006. Comparative genomics of multidrug resistance in Acinetobacter baumannii. PLoS Genet 2:e7. https://doi.org/10.1371/journal.pgen.0020007. Download Table S3, PDF file, 0.03 MB. Copyright © 2022 Godeux et al. 2022 Godeux et al. https://creativecommons.org/licenses/by/4.0/ This content is distributed under the terms of the Creative Commons Attribution 4.0 International license.

**FIG 5:**
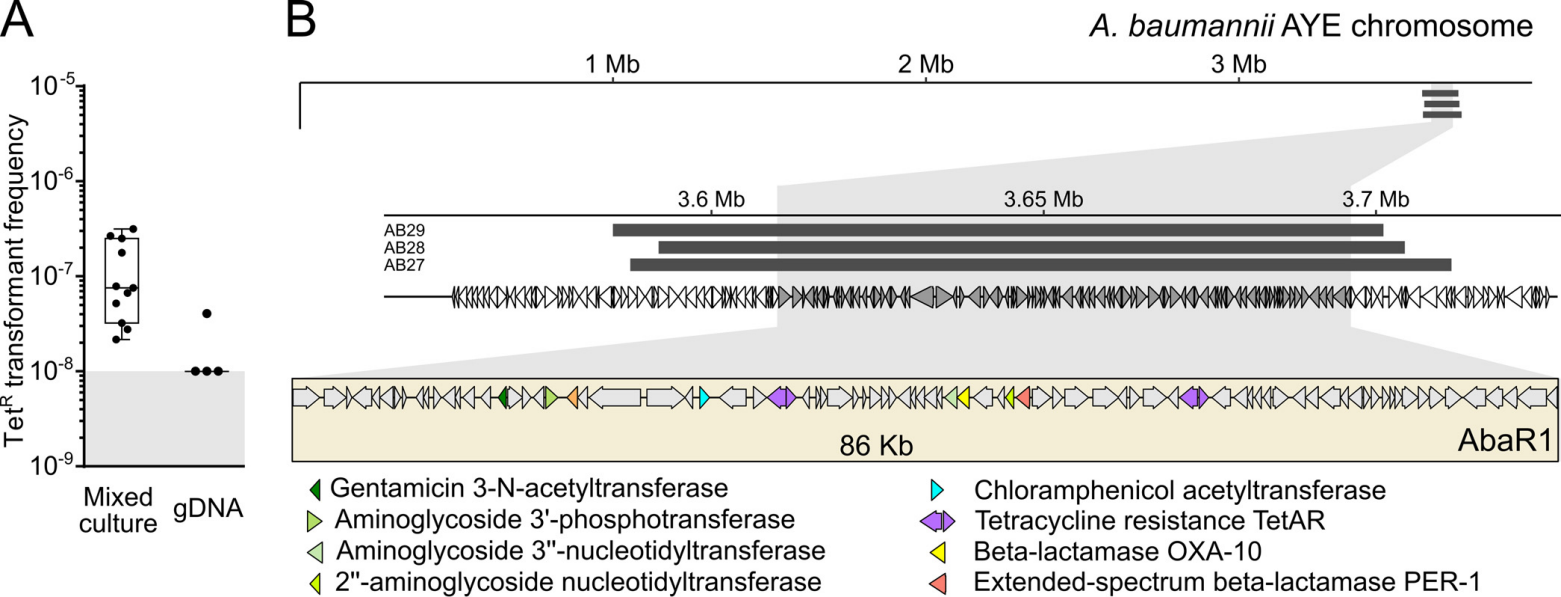
Genomic analysis of the acquisition of the AbaR1 island. (A) Transformation frequencies of the acquisition of tetracycline resistance carried by the AbaR1 island of the AYE strain, in a mixed-culture setup between the AYE strain impaired for natural transformation (*comEC*::*aacC4*) and the M2 Rif^r^ strain and by supplying M2 Rif^r^ with genomic DNA extracted from AYE. (B) Graphical representation of the chromosome of the AYE strain (thin black line) and location of the acquired DNA fragments (light gray thick lines) by transformants of the A. nosocomialis strain M2 during mixed culture with AYE. Bottom diagram, close-up view of the acquired fragments and the AbaR1 island. The genomes of tetracycline-resistant recombinants of M2 were sequenced. Variant calling and identification of converted markers (SNPs of AYE acquired by M2) were used to delineate the acquired regions (see Materials and Methods).

The AbaR4 island is one of the many representatives of resistance islands that can be found in A. baumannii clinical isolates (9). The largest resistance island described so far, the 86-kb-long AbaR1, is found in the AYE strain (8). We thus tested whether this island could be acquired by the M2 strain. In a previous work, we showed that the AbaR1 island conferred resistance to aminoglycosides, tetracycline, and some beta-lactams (35). We thus used tetracycline resistance as a marker for the transfer of AbaR1 from A. baumannii strain AYE (donor strain) to A. nosocomialis strain M2 (recipient strain). To test the acquisition of AbaR1 by M2, we used the rifampicin-resistant derivative of strain M2 and a derivative of the strain AYE impaired for natural transformation (Δ*comEC*::*aacC4*). The mixed-culture condition resulted in recombinants emerging at a frequency of about 10^−7^ (Fig. 5A). It is noteworthy that only few transformants were detected when using purified gDNA, confirming that the mixed culture brought together conditions favorable to the transfer of the large resistance island (Fig. 5A). The resulting transformants displayed the resistance profile expected from the known resistance associated with AbaR1 (Table S2), indicating that the entire 86-kb-long island had indeed been acquired. Genome sequencing of three tetracycline-resistant transformants confirmed that the entire AbaR1 island (86 kb) was inserted into the genome of the recipient cells (Fig. 5B and Fig. S7). The recombination tracts flanking the *comM* locus indicated that the imported DNA molecules ranged from 112 to 123 kb (Fig. 5B and Fig. S7). Taken together, these results demonstrate that recombinants can emerge from the encounter of Acinetobacter isolates, even if their genomes display relatively low sequence identity (90%). The interbacterial transfer can result in the efficient recombination of homologous regions and the acquisition of large heterologous segments up to 86 kb, such as AbaR islands conferring resistance to multiple antibiotics, including carbapenems.

10.1128/mBio.02631-21.7 FIG S7 Genomic analysis of the acquisition of the AbaR1 island. Graphical representation of the acquired fragments and the AbaR1 island in the M2 transformants. Variant calling and identification of converted markers (SNPs of AYE acquired by M2) were used to delineate the acquired regions (see Materials and Methods). Thin green lines represent coverage, and the outermost right scale indicates the results of read screening corresponding to the AbaR sequence in recombinants. Orange triangles represent individual SNPs used to determine recombination tracts. Download FIG S7, TIF file, 0.2 MB. Copyright © 2022 Godeux et al. 2022 Godeux et al. https://creativecommons.org/licenses/by/4.0/ This content is distributed under the terms of the Creative Commons Attribution 4.0 International license.

## DISCUSSION

To gain insight into the mechanism leading to emergence of carbapenem and more broadly multidrug resistance in pathogenic Acinetobacter spp., we devised an experimental system to monitor emergence of recombinant progeny and diffusion of antibiotic resistance between multiple combinations of pathogenic Acinetobacter strains. We observed that resistance is efficiently acquired within mixed populations of A. baumannii strains and even between A. baumannii and A. nosocomialis. Investigating in more detail a specific mixed population, we demonstrated that rapid gene transfer occurs through natural transformation in communities of sessile cells, leading to antibiotic resistance. Resistance genes were either on a composite transposon (Tn*2006* carrying *bla*_OXA-23_) or on large resistance islands (AbaR4 and AbaR1). So far, demonstrations of gene acquisition by natural transformation in A. baumannii and A. nosocomialis were obtained using purified DNA (23–28). Our results indicate that this form of DNA is a poor substrate for transformation in comparison to the DNA released in the extracellular milieu during growth or upon bacterial interaction. DNA purification is known to lead to some DNA degradation, with nicks which will terminate the import of the single-stranded DNA (ssDNA) (38). Mixed culture allowed a transfer of up to 123 kb from strain A. baumannii AYE to A. nosocomialis, corresponding to over 3% of the recipient strain’s genome. Our results corroborate seminal observations in Streptococcus pneumoniae, in which mixed sessile populations were found to support larger recombination events (up to 29 kb) (39). Similarly, interaction of V. cholerae strains also promotes transfer of a large genomic region (up to 168 kb) (40). Importantly, our experiments recapitulate the observed recombination events associated with the acquisition by A. baumannii strains of AbGRI3 and AbGRI5 from other strains of the global clone 2 (GC2) lineage (14, 15). Acquisition by natural transformation would therefore explain the acquisition of AbaRs described for GC2 strains (41) but also for other Acinetobacter species from the A. baumannii-A. calcoaceticus complex, namely, A. nosocomialis, A. haemolyticus, A. pittii, and A. seifertii (9, 42, 43). Our experimental observation leads us to reconsider the role of natural transformation in the acquisition of large islands of resistance by A. baumannii previously attributed to generalized transduction based on presumed limited size of acquisition enabled by natural transformation (20). Transfer of a very large resistance island such as AbaR1 is less frequent, as the odds of importing an intact DNA are likely lower for long DNA fragments. Moreover, acquisition of new genetic material has a potential cost to bacterial replication (44). In the specific case of AbaR1, importing numerous antibiotic and heavy metal resistance genes may be detrimental for the recipient strain, with a temporary fitness cost that would affect the apparent transformation frequency (45, 46). Another consequence of acquisition by natural transformation of these genomic islands is that their horizontal transfer would be conservative of their insertion site. Genomics studies suggests that Ab-RI were initially introduced into A. baumannii through plasmid conjugation, with subsequent insertion in the chromosome (12, 13). Ab-RI retain the features of ancestral Tn*7* elements (47), and although transposition activity tested with AbaR1 was not detectable (48), Ab-RI are found inserted in over 50 chromosomal insertion sites (9). Yet the vast majority of Ab-RI appear to be inserted in a specific locus, the *comM* gene (9, 11, 12), which encodes a putative helicase involved in natural transformation (35, 49, 50). Supported by genomic evidence (14, 15), our results suggest that HGT of Ab-RI can occur through homologous recombination produced by natural transformation. This mechanism of HGT would propagate the inactivation of *comM* and the apparent fixation of some Ab-RI at this specific locus.

We investigated the role of the T6SS in the transfer of resistance upon bacterial interaction. The competition mechanism was an evident culprit to trigger the release of DNA from prey cells, as described for A. baylyi and V. cholerae (31, 32, 40). Although we found that the T6SS is partially involved in acquisition of resistance genes, we found that T6SS-mediated killing is potentially impaired under conditions compatible with natural transformation. Our results corroborate previous findings that T6SS development is dependent on growth conditions, with the observation of a reduced T6SS activity in minimal medium for A. nosocomialis M2 as determined by Hcp secretion (51). Contribution of the T6SS to DNA release and diffusion of resistance determinants may, however, depend on strain combination, as T6SS activity was shown to be highly dependent on the strains in competitions (36, 52, 53). In addition, our results show that the direct negative interaction between cells is not the sole mechanism promoting release of DNA and subsequent acquisition of the resistance determinant. We found that the contact-independent release of DNA during growth is sufficient to mediate high rates of acquisition of resistance through natural transformation. In addition to passive release by autolysis upon cell death, several mechanisms of active DNA release in the extracellular compartment had been described for other transformable species. In Neisseria gonorrhoeae, genomic DNA is actively secreted through a type IV secretion system by the donor strain, delivering a more potent substrate of transformation than DNA released upon autolysis (54, 55). In Pseudomonas aeruginosa, DNA release is dependent on a cryptic prophage endolysin that is required for formation of biofilm, where transformation can also occur (56–58). Indeed, extracellular DNA is also a critical component of the biofilm matrix (59), and treatment with DNase leads to severe biofilm alteration for several pathogenic bacteria, including A. baumannii (60). However, as suggested by our results, it is conceivable that the level of DNA release may vary between strains of A. baumannii and thereby influence biofilm formation, the diffusion of resistance genes, and their acquisition by neighboring cells.

Altogether, this work highlights the importance of one of the major HGT mechanisms in bacteria, natural transformation, in the acquisition by A. baumannii of resistance to clinically relevant antibiotics. By naturally releasing high-quality DNA in their environment, some strains of A. baumannii may be highly potent donors for the uptake and recombination of large DNA fragments by natural transformation. This mechanism may enable a rapid and direct acquisition of multiple antibiotic resistance genes by A. baumannii and explain the evolution by recombination observed at the genomic scale.

## MATERIALS AND METHODS

### Bacterial strains and growth conditions.

The bacterial strains or strains used in this study are listed in Table S1. Acinetobacter baumannii isolates and strains were grown in lysogeny broth (LB; Lennox) or tryptone-NaCl medium (5 g/L of tryptone, 2.5 g/L NaCl). All experiments were performed at 37°C. Antibiotic concentrations were 15 μg/mL for tetracycline, 50 μg/mL for kanamycin, 1.6 μg/mL for imipenem (always spread extemporaneously on an LB agar plate), and 100 μg/mL for rifampicin.

10.1128/mBio.02631-21.8 TABLE S1 Bacterial strains, plasmids, and primers used in this study. Download Table S1, PDF file, 0.1 MB. Copyright © 2022 Godeux et al. 2022 Godeux et al. https://creativecommons.org/licenses/by/4.0/ This content is distributed under the terms of the Creative Commons Attribution 4.0 International license.

### Construction of bacterial strains.

All the oligonucleotides used in this study for genetic modification are listed in Table S1. Gene disruptions were performed using a scarless genome editing strategy described previously (35). The oligonucleotides used for strain construction are listed in Table S1. Rifampicin-resistant derivatives were obtained by selecting spontaneous mutants from an overnight culture in liquid LB on LB agar supplemented with rifampicin.

### Detection of recombinants in mixed cultures.

Isolates to be tested for the production of recombinants were grown overnight on LB agar plates and then inoculated and grown in 2 mL of LB until the cultures reach an optical density at 600 nm (OD_600_) of 1. The bacterial broths were then diluted to an OD_600_ of 0.01 in phosphate-buffered saline (PBS). Then equal volumes of bacterial suspensions were mixed by pairs, each consisting of an imipenem-resistant (Imi^r^) and rifampicin-resistant (Rif^r^) isolate. The mixture (2.5 μL) was deposited on the surface of 1 mL of tryptone-NaCl medium solidified with 2% agarose D3 (Euromedex) poured into 2-mL microtubes or in wells of 24-well plates and incubated overnight at 37°C. Bacteria were then resuspended in PBS and were plated onto LB agar plates without antibiotics and LB agar plates containing rifampicin (100 μg/mL) and imipenem (1.6 μg/mL) with either beads or easySpiral Pro (Interscience). Recombinant frequencies were determined through calculation of the ratio of the number of CFU on rifampicin and imipenem plates to the total number of CFU on plates without antibiotics.

### Transformation assay.

Transformation assays were performed as previously described (35) except that then bacterial suspensions of the recipient strain were mixed with an equal volume of either genomic DNA extracted from the donor strain (concentration, 200 ng/μL), a bacterial suspension of the donor strain at an OD_600_ of 0.01 for mixed culture, or a filtered spent medium. DNase I treatment was performed at a final concentration of 0.3 U/μL (Sigma). All the transformation assays were performed on at least two separate occasions with three independent transformation reactions (three different bacterial cultures). All the independent data points are plotted. For kinetics of transformation, bacteria were recovered at indicated times points with bacteria harvested from an independent sample and plated as previously described.

### DNA measurement in spent media.

Supernatants were obtained by centrifugation (3,000 × *g*, 3 min) of a culture of the donor strain for 4 h in 37°C tryptone-NaCl liquid medium with agitation at 200 rpm. The supernatant was then filtered (0.22-μm-size pore filter). DNA quantification was performed by fluorescent labeling with Quant-iT reagent (Invitrogen Qubit dsDNA HS assay kit; Thermo Fisher Scientific) measured with Infinite M200 PRO (TECAN) and Magellan 7.1 SP1 (TECAN).

### Genome sequencing, assembly, and annotations.

The genome of 40288 was sequenced using Illumina technology (paired-end, read length 100 nt, HiSeq instrument) and Oxford Nanopore technology (rapid barcoding kit, MinION instrument) with a read coverage of 100×. Short (Illumina) and long (Oxford Nanopore) reads were used to assemble the genome with Unicycler (61). This generated a complete genome consisting of a circular chromosome of 4,084,922 bp and circular plasmid of 145,711 bp. Genes were annotated with Prokka (62), using the A. baumannii AB5075-UW genome as a reference (63). The genome of the human clinical isolate CNRAB1 was sequenced using similar procedures.

### Variant calling and detection of recombination events.

Genomes were sequenced on a NovaSeq instrument (Illumina, 2 × 150-bp paired end). Reads were subsampled to 50× coverage of the donor genome using *seqtk* version 1.3-r106 (https://github.com/lh3/seqtk). Reads of recombinants were mapped to their corresponding donor genome (M2, 40288, or AYE) using *bwa mem* version 0.7.17-r1888 (64) and sorted using *samtools* version 1.10.3 (65). Donor (40288 or AYE) and recipient (M2) genome short reads were simulated using *wgsim* version 1.10 (https://github.com/lh3/wgsim) and mapped to the donor genome (40288 or AYE) using the same parameters as for transformant sequencing reads. Reads were then tallied on the donor genome using *bcftools* version 1.10, to keep only sites with read coverage above 25×, where haplotype differs between donor and recipient and match donor haplotype. The variant calling and identification of converted markers were performed using *twgt* version 0.1.0.5 (https://gitlab.com/bacterial-gbgc/twgt/-/tree/master). The resulting VCF file was then parsed using *VariantAnnotation* (66) using R version 3.6.6. With Tn*2006*, AbaR4, and AbaR1 being absent from the recipient genome, their insertions in the transformant genome were confirmed via read screening: their sequence together with 1 kb of flanking sequence was sketched using *mash sketch* and the presence of this sketch in sequencing reads was confirmed using *mash screen* version 2.2.2 (67).

### Data availability.

Genome sequences are available within BioProject PRJNA741866. Raw sequencing reads are available in Sequence Read Archive (SRA) within PRJNA741866.
