# Erratum for Godeux et al., “Interbacterial Transfer of Carbapenem Resistance and Large Antibiotic Resistance Islands by Natural Transformation in Pathogenic Acinetobacter”

**DOI:** 10.1128/mbio.00526-22

**Published:** 2022-03-14

**Authors:** Anne-Sophie Godeux, Elin Svedholm, Samuel Barreto, Anaïs Potron, Samuel Venner, Xavier Charpentier, Maria-Halima Laaberki

**Affiliations:** a Centre International de Recherche en Infectiologie (CIRI), INSERM, U1111, Université Claude Bernard Lyon 1, CNRS, UMR5308, École Normale Supérieure de Lyon, Université de Lyon, Villeurbanne, France; b Université de Lyon, VetAgro Sup, Marcy l’Etoile, France; c UMR CNRS 5558, Laboratoire de Biométrie et Biologie Évolutive (LBBE), Université Claude Bernard Lyon 1, Villeurbanne, France; d French National Reference Center for Antibiotic Resistance, University Hospital of Besançon, Besançon, France; e UMR6249, CNRS Chrono-Environnement, Franche-Comté University, Besançon, France

## ERRATUM

Volume 13, no. 1, e02631-21, 2022, https://doi.org/10.1128/mbio.02631-21. Elin Svedholm’s last name was spelled incorrectly in the original article. The byline should appear as shown above.

