# Supplementary material for: Interbacterial Transfer of Carbapenem Resistance and Large Antibiotic Resistance Islands by Natural Transformation in Pathogenic Acinetobacter
Source: mBio. 2022 Jan 25;13(1):e02631-21. doi: 10.1128/mbio.02631-21 (PMC8787482; doi:10.1128/mbio.02631-21)
Supplement: TABLE S2 [file mbio.02631-21-st002.pdf]

| | Minimum inhibitory concentration (E-test)[a] | | | |
|---|---|---|---|---|
| Antibiotic | 40288 WT | M2 WT | M2 Rif$^R$ *comM::*AbaR4 | M2 Rif$^R$ *vgrG3::*Tn*2006* |
| IMP | >32 | 0.25 | >32 | >32 |

| | Inhibition zone diameter in mm [b] | | | | |
|---|---|---|---|---|---|
| Antibiotic | AYE WT | M2 WT | M2 Rif$^R$ | M2 Rif$^R$ *comM::*AbaR1 Clone 1 | M2 Rif$^R$ *comM::*AbaR1 Clone 2 |
| AMK | 8 | 29 | 28 | 15 | 14 |
| GEN | 6 | 29 | 28 | 12 | 12 |
| TOB | 6 | 26 | 25 | 9 | 7 |
| TIG | 19 | 25 | 27 | 29 | 27 |
| FOS | 8 | 6 | 6 | 6 | 6 |
| COL | 19 | 19 | 20 | 20 | 20 |
| CIP | 6 | 26 | 29 | 29 | 29 |
| PIP | 6 | 22 | 22 | 12 | 14 |
| TIC | 6 | 30 | 25 | 6 | 6 |
| IMP | 28 | 40 | 40 | 40 | 40 |
| TZP | 14 | 29 | 25 | 25 | 25 |
| TIM | 15 | 29 | 28 | 23 | 25 |
| MEM | 20 | 33 | 27 | 23 | 24 |
| CAZ | 6 | 23 | 22 | 6 | 6 |
| FEP | 6 | 26 | 25 | 6 | 6 |
| ATM | 6 | 16 | 10 | 6 | 6 |
| RIF | 13 | 17 | 6 | 6 | 6 |

| | Minimum inhibitory concentration (E-test) | | | | |
|---|---|---|---|---|---|
| Antibiotic | M2 | AYE WT | AYE ∆AbaR[c] | M2 Rif$^R$ *comM::*AbaR1 Clone 1 | M2 Rif$^R$ *comM::*AbaR1 Clone 2 |
| TET | 4 | 128 | 8 | 48 | 48 |

## Table S2: Antimicrobial susceptibility profiles of mixed culture transformants

[a] E-test strips (bioMérieux, France) were used to compare resistance to imipenem or tetracycline.

[b] Amikacin (AMK), gentamicin (GEN), tobramycin (TOB), tigecycline (TIG), Fosfomycin (FOS), colistin (COL), ciprofloxacin (CIP), piperacillin (PIP), piperacillin-tazobactam (TZP), ticarcillin (TIC), ticarcillin-clavulanic acid (TIM), ceftazidime (CAZ), cefepime (FEP), meropenem (MEM), aztreonam (ATM), rifampin (RIF) and imipenem (IMP). Susceptibilities were evaluated as by disc diffusion on Muller-Hinton agar (Biorad, France) as in (1)

[c] AYE ∆AbaR correspond to AbaR-cured AYE derivative (1). The strain *Pseudomonas aeruginosa* CIP 7110 was used as control for all the antibiotic susceptibility testing.

(1) Godeux A-S, Svedholm E, Lupo A, Haenni M, Venner S, Laaberki M-H, Charpentier X. 2020. Scarless Removal of Large Resistance Island AbaR Results in Antibiotic Susceptibility and Increased Natural Transformability in Acinetobacter baumannii. Antimicrob Agents Chemother 64.
