# Supplementary material for: Interbacterial Transfer of Carbapenem Resistance and Large Antibiotic Resistance Islands by Natural Transformation in Pathogenic Acinetobacter
Source: mBio. 2022 Jan 25;13(1):e02631-21. doi: 10.1128/mbio.02631-21 (PMC8787482; doi:10.1128/mbio.02631-21)
Supplement: TABLE S3 [file mbio.02631-21-st003.pdf]

Sample	Donor strain	Loci	First acquired SNP[a]	Last acquired SNP[b]	Length of imported DNA (bp)[c]
AB25	40288	Tn*2006*	91048	148295	57247
AB24	40288	Tn*2006*	120337	151404	31067
AB23	40288	Tn*2006*	109565	128246	18681
AB22	40288	Tn*2006*	120849	133850	13001
AB21	40288	Tn*2006*	101695	157494	55799
AB20	40288	Tn*2006*	71139	148223	77084
AB19	40288	Tn*2006*	120442	139003	18561
AB26	40288	AbaR4	3815631	3901652	86021
AB18	40288	AbaR4	3813832	3858827	44995
AB17	40288	AbaR4	3766851	3854677	87826
AB16	40288	AbaR4	3785214	3854677	69463
AB15	40288	AbaR4	3814000	3853045	39045
AB14	40288	AbaR4	3802492	3857180	54688
AB13	40288	AbaR4	3813642	3840698	27056
AB12	40288	AbaR4	3770779	3860715	89936
AB29	AYE	AbaR1	3585163	3701113	115950
AB28	AYE	AbaR1	3592041	3704339	112298
AB27	AYE	AbaR1	3587780	3711356	123576

Table S3. Estimated length of the imported DNA molecules leading to the detected recombination tracts.

SNPs acquired by the M2 recipient strain were mapped onto the donor genome and position of the first[a] and last[b] acquired provide an estimate of the length of the imported DNA molecule[c] that resulted in the observed recombination tracts.
