# Supplementary material for: Interbacterial Transfer of Carbapenem Resistance and Large Antibiotic Resistance Islands by Natural Transformation in Pathogenic Acinetobacter
Source: mBio. 2022 Jan 25;13(1):e02631-21. doi: 10.1128/mbio.02631-21 (PMC8787482; doi:10.1128/mbio.02631-21)
Supplement: TABLE S1 [file mbio.02631-21-st001.pdf]

Table S1: Bacterial strains, plasmids and primers used in this study.

**Strain**	**Description/phenotype**	**Reference**
Recipient strains for natural transformation		
M2	*A. nosocomialis* strain M2 wild-type	(1)
M2 Rif^R	Spontaneous *rpoB* mutant; rifampicin resistant	This study
M2 Rif^R Δ*comEC::aacC4*	Insertion of an apramycin resistance cassette in the *comEC* gene of the M2 Rif^R strain; Non transformable and rifampicin resistant	This study
M2 Rif^R Δ*recA::aacC4*	Insertion of an apramycin resistance cassette in the *recA* gene of the M2 Rif^R strain; Non transformable and rifampicin resistant	This study
M2 Rif^R Δ*pilA::kan*	Insertion of a kanamycin resistance cassette in the *pilA* gene of the M2 Rif^R strain; Non transformable and rifampicin resistant	This study
AYE	*A. baumannii* strain AYE wild-type	(2)
AYE Rif^R	Spontaneous *rpoB* mutant; rifampicin resistant	This study
29D2	*A. baumannii* nonclinical isolate 29D2 wild-type	(3)
29D2 Rif^R	Spontaneous *rpoB* mutant; rifampicin resistant	This study
A118	*A. baumannii* strain A118 wild-type	(4)
A118 Rif^R	Spontaneous *rpoB* mutant; rifampicin resistant	This study
27304	*A. baumannii* veterinary clinical isolate 27304	Resapath, ANSES
27304 Rif^R	Spontaneous *rpoB* mutant; rifampicin resistant	This study
27024	*A. baumannii* veterinary clinical isolate 27024	Resapath, ANSES
27024 Rif^R	Spontaneous *rpoB* mutant; rifampicin resistant	This study

Donor strains for natural transformation		
40288	*A. baumannii* veterinary clinical isolate 40288; carbapenem resistant	(5)
40288 Δ*comEC::aacC4*	Insertion of an apramycin resistance cassette in the *comEC* gene; Not naturally transformable	This study
AB5075	*A. baumannii* strain AB5075 wild-type; carbapenem resistant	(6)
37986	*A. baumannii* veterinary clinical isolate 37986; carbapenem resistant	(5)
CNRAB1	*A. baumannii* human clinical isolate CNRAB1; carbapenem resistant	Centre National de Référence de l'antibiorésistance de Besançon, France.
M2 Δ*comEC::aacC4*	Insertion of an apramycin resistance cassette in the *comEC* gene; Not naturally transformable	This study
AYE Δ*comEC::aacC4*	Insertion of an apramycin resistance cassette in the *comEC* gene; Not naturally transformable	This study
AB5075 Δ*comEC::aacC4*	Insertion of an apramycin resistance cassette in the *comEC* gene; Not naturally transformable	This study

Genetic construct	Name	Primer Sequence 5' to 3'	Template; Annealing site
Chromosomal modifications using overlap extension PCR			
comEC::aacC4	comEC_UP_F	TGGTGTCGCTGTACTAATTACGGT	*Acinetobacter* genomic DNA; 2kb upstream of the *comEC* gene
	comEC_apra_R	GTGCCGTGCTAGTAGCACGCCCTCCCGTTCAGAGCCTCGCCCACTTTTTC	*Acinetobacter* genomic DNA; Chimeric, middle of the *comEC* gene
	comEC_apra_F	GGCGAGCGGTCAGCTAACCGACTCGAGTACTGGATAGGCTCTGGTTGAGTA	*Acinetobacter* genomic DNA; Chimeric, middle of the *comEC* gene
	Apr_Fw	CAGGCTGGGTGCCAAGCTCT	pMHL-2 (Godeux et al., 2018) ; downstream *aacC4* gene
	Apr_Rev	TCATGAGCTCAGCCAATCGACTGG	pMHL-2 (Godeux et al., 2018) ; upstream

			aacC4 gene
	comEC_DW_R	CTTCAGTTCACGCATCAAGCTTGT	*Acinetobacter* genomic DNA; 2kb upstream of the *comEC* gene
pilA::kan	asg-145	GTTATGGCGGCGGTGGAGGTC	*Acinetobacter* genomic DNA ; 1kb upstream of the *pilA* gene
	asg-143	GAACACTTGAGGCGCAGAGTAAATGCTTCAGATGC	*Acinetobacter* genomic DNA ; Chimeric, middle of the *pilA* gene
	asg-144	CCTTCTTCACGAGGCATCAATCATTTGTAGCATCG	*Acinetobacter* genomic DNA ; Chimeric, middle of the *pilA* gene
	kan-F	CTGCGCCTCAAGTGTTC	KanR gene cassette ; upstream the *kanR* gene
	kan-R	TGCCTCGTGAAGAAGG	KanR gene cassette ; downstream the *kanR* gene
	asg-146	AGAATACTTGCATAGTGACAGGTTACAG	*Acinetobacter* genomic DNA ; 1kb downstream of the *pilA* gene
recA::aacC4	LH114_V2_recA	CTTTTTCATTGCTCTCAAGATGAAC	*Acinetobacter* genomic DNA; 2kb upstream of the *recA* gene
	LH115_V2_recA	GGAACTTCGAAGCAGCTCCAGCCTACACAATCGTAATGCTTTGCTTTTATTCT CATCC	*Acinetobacter* genomic DNA; Chimeric: 5’ of the *recA* gene and upstream of *aac4* gene
	LH116_recA_AB 5075	AACTAAGGAGGATATTCATATGGACCATGGCGACTACAGGCACAAATGGTGC	*Acinetobacter* genomic DNA; Chimeric: downstream of *aac4* gene and 3’ of the *recA* gene
	LH117_recA_AB 5075	GTTGGTACAATGCTCGAGGTG	*Acinetobacter* genomic DNA; 2kb downstream of the *recA* gene
Primers for control of the transformants using colony PCR			
	comM-For	CCACAATGGAACAAGAAGATGTCT	M2 genomic DNA; 5’ of the *comM* gene
	comM-Rev	TTAAGAGTGATTACCTCGATAAGA	M2 genomic DNA; 3’ of the *comM* gene

	OXA23-For	GATCGGATTGGAGAACCAGA	*bla*$_{OXA-23}$ gene
	OXA23-Rev	ATTTCTGACCGCATTTCCAT	*bla*$_{OXA-23}$ gene
	mlo-80	CGGATCTTCGATGCTGGC	AbaR1 from AYE strain
	mlo-84	GCAACGATGTTACGCAGC	AbaR1 from AYE strain
	5'-J-Rev	ATGGAATGTAGTACTCTGACG	AbaR1 from AYE; AbaR junction
	3'-J-For	GATTCACATCATATTCATTCGCCC	AbaR1 from AYE; AbaR junction

1. Niu C, Clemmer KM, Bonomo RA, Rather PN. 2008. Isolation and Characterization of an Autoinducer Synthase from *Acinetobacter baumannii*. J Bacteriol 190:3386–3392.
2. Fournier P-E, Vallenet D, Barbe V, Audic S, Ogata H, Poirel L, Richet H, Robert C, Mangenot S, Abergel C, Nordmann P, Weissenbach J, Raoult D, Claverie J-M. 2006. Comparative genomics of multidrug resistance in *Acinetobacter baumannii*. PLoS genetics 2:e7.
3. Wilharm G, Skiebe E, Higgins PG, Poppel MT, Blaschke U, Leser S, Heider C, Heindorf M, Brauner P, Jäckel U, Böhland K, Cuny C, Łopińska A, Kaminski P, Kasprzak M, Bochenski M, Ciebiera O, Tobółka M, Żołnierowicz KM, Siekiera J, Seifert H, Gagné S, Salcedo SP, Kaatz M, Layer F, Bender JK, Fuchs S, Semmler T, Pfeifer Y, Jerzak L. Relatedness of wildlife and livestock avian isolates of the nosocomial pathogen *Acinetobacter baumannii* to lineages spread in hospitals worldwide. Environ Microbiol 19:4349–4364.
4. Ramirez MS, Don M, Merkier AK, Bistué AJS, Zorreguieta A, Centrón D, Tolmasky ME. 2010. Naturally Competent *Acinetobacter baumannii* Clinical Isolate as a Convenient Model for Genetic Studies. J Clin Microbiol 48:1488–1490.
5. Lupo A, Châtre P, Ponsin C, Saras E, Jean-Boulouis H, Keck N, Haenni M, Madec J-Y. 2016. Clonal Spread of bla OXA-23 ST25 *Acinetobacter baumannii* in Companion Animals in France. Antimicrobial Agents and Chemotherapy AAC.01881-16.
6. Jacobs AC, Thompson MG, Black CC, Kessler JL, Clark LP, McQueary CN, Gancz HY, Corey BW, Moon JK, Si Y, Owen MT, Hallock JD, Kwak YI, Summers A, Li CZ, Rasko DA, Penwell WF, Honnold CL, Wise MC, Waterman PE, Lesho EP, Stewart RL, Actis LA, Palys TJ, Craft DW, Zurawski DV. 2014. AB5075, a Highly Virulent Isolate of *Acinetobacter baumannii*, as a Model Strain for the Evaluation of Pathogenesis and Antimicrobial Treatments. mBio 5:e01076-14.
